# miR-330 Regulates Colorectal Cancer Oncogenesis by Targeting BACH1

**DOI:** 10.34172/apb.2020.054

**Published:** 2020-05-11

**Authors:** Solmaz Shirjang, Behzad Mansoori, Ali Mohammadi, Neda Shajari, Pascal H.G. Duijf, Souzan Najafi, Fereydoon Abedi Gaballu, Katayoon Nofouzi, Behzad Baradaran

**Affiliations:** ^1^Immunology Research Center, Tabriz University of Medical Sciences, Tabriz, Iran.; ^2^Student Research Committee, Tabriz University of Medical Sciences, Tabriz, Iran.; ^3^Department of Cancer and Inflammation Research, Institute for Molecular Medicine, University of Southern Denmark, Odense, Denmark.; ^4^Department of Immunology, School of Medicine, Shiraz University of Medical Science, Shiraz, Iran.; ^5^University of Queensland Diamantina Institute, The University of Queensland, Translational Research Institute, Brisbane, Australia.; ^6^Department of Pathobiology, Faculty of Veterinary Medicine, University of Tabriz, Tabriz, Iran.

**Keywords:** miR-330, BACH1, MMP9, CXCR4, VEGFR, Proliferation, Colorectal cancer

## Abstract

***Purpose:*** Based on WHO report, colorectal cancer (CRC) is the second cause of death among patients with cancer worldwide. Dysregulation of miRNAs expressions has been demonstrated in different human cancers, especially CRC. Studies have shown that miR-330 could act as both TS-miR and/or oncomiR in different types of cancers. BACH1 is also identified as a transcription factor, which is involved in ontogenesis. In this study, we evaluated the CRC suppression via silencing of BACH1 by small silencer molecule called miR-330.

***Methods:*** Firstly, we analyzed the BACH1, miR-330-3p and miR-330-5p expressions according to the colon adenocarcinoma (COAD) and rectal adenocarcinoma (READ) project established from a patient of the colon and rectal cancer patients in The Cancer Genome Atlas (TCGA) database. The targeting of BACH1 via miR-330 in human CRC cells was evaluated by Vejnar bioinformatics methods, and confirmed by qRT-PCR and western blot analysis. Proliferation was performed by MTT assay. The MMP9, CXCR4, and VEGFR proteins were measured by western blotting.

***Results:*** The analysis of BACH1, miR-330-3p, and miR-330-5p expressions according to the COAD and READ projects showed that BACH1 was overexpressed, but miR-330-3p and miR330-5p were reduced in CRC tumors compared to normal controls. The miR-330 induction prevented proliferation of CRC cell by targeting BACH1 mRNA, which represses MMP9, C-X-C chemokine receptor type 4 (CXCR4), and vascular endothelial growth factor receptor (VEGFR) proteins expressions.

***Conclusion:*** Our results suggested that BACH1 is a potential target for miR-330 in CRC cells. The miR-330 induction inhibits CRC cells proliferation by suppressing BACH1 expression in posttranscriptional level. It was suggested that targeting of BACH1 via miRNA such as miR-330 could be a valid strategy in the field of CRC targeted therapy via modulating the oncogenic signaling pathway.

## Introduction

For both sexes combined, colorectal cancer (CRC) is the third most commonly diagnosed cancer (1.8 million new cases).^1^ Due to the lack of early detection and effective therapeutic strategy in CRC patients, their survival rate has been limited.^2^ Inadequate knowledge about the molecular mechanisms of CRC impedes the improvement of potential diagnostic, prognostic, and therapeutic targets. Different gene-expression signatures are available for cancer prognosis or clinical outcomes; however, recent evidence has shown that microRNAs mainly control various types of carcinogenesis-related genes.^3^

MicroRNAs (miRNAs) are small single strand noncoding RNAs containing 19-22 nucleotides that are transcribed from introns or non-coding DNA, and involved in the translational inhibition or silencing of their target messenger RNAs (mRNAs) via binding to the complementary sites in their three prime untranslated regions (3’-UTR).^4^

Calin et al displayed the first connection between microRNA and cancer. They found that the miR-15a and miR-16-1 are reduced in B-cell chronic lymphocytic leukemia (B-CLL). They suggested that these two microRNAs have a potential role as tumor suppressors.^5^ In 2003, only one-year later, Michael et al published the earliest report which identified dysregulation of miRNAs in CRC compared to normal colorectal cells. They discovered a reduction of both miR-143 and miR-145 in pre-cancerous and neoplastic colorectal tissues compared to the normal samples.^6^ More and more miRNAs have been found to be deregulated in CRC samples since their study, such as miR-15b, miR-20, miR-21, miR-31, miR-181b, miR-191, miR-192, and miR-200c.^7-13^ Ruike et al investigated 16 human cell lines and 155 types of mature miRNA including miR-330. They showed that the level of mature miR-330 was significantly decreased in most of the cancer cell lines.^14^ Lee et al demonstrated that miR-330 has significantly lower expression in prostate cancer cells than the normal cells and has a reverse correlation with E2F1 in prostate clinical samples. Also, they revealed its pro-apoptotic role through reducing Akt phosphorylation by targeting E2F1.^15^ In another study, Hodzic et al revealed that miR-330 was reduced in colorectal tissues compared to the normal tissue samples.^16^

BACH1 (BTB and CNC homolog 1) is a transcription regulator protein and it maps to 21q21.3. It is widely expressed in mammalian tissues, and the human variant consists of 736 amino acids.^17^ This transcription factor regulates oxidative stress and subsequently induces cellular senescence via induction of heme oxygenase-1 (HO-1).^18^ Recently, growing evidence has revealed the role of BACH1 in tumor initiation, progression, and metastasis. Yun et al reported that BACH1 is a let-7-regulated transcription factor which induces the expression of matrix metalloproteinases 1 (MMP1) in favor of tumor progression.^19^ Moreover, Liang et al demonstrated that BACH1 knockdown resulted in significant suppression of metastasis, and conversely, ectopic expression of BACH1 was significantly linked to the malignancy of breast cancer cells. They introduced BACH1 involved as a major regulator protein of breast cancer to bone metastasis.^20^ Shajari et al have recently demonstrated that overexpression of BACH1 plays an important role in the carcinogenesis and metastasis of prostate cancer. They found a direct correlation between the expression of BACH1 and EMT-related factors such as high mobility group A2 (HMGA2), let-7a, C-X-C chemokine receptor type 4 (CXCR4), vimentin, MMP1, MMP9, and MMP13.^21-23^

The aim of this study was to demonstrate the possible change in miR-330 expression in CRC tissues compared to normal controls. We also showed that miR-330 could target BACH1 expression and cause CRC cells proliferation and migration suppression via repressing EMT related proteins including MMP9, CXCR4, and vascular endothelial growth factor receptor (VEGFR).

## Materials and Methods

### The miR-330 and BACH1 expression and survival analysis in CRC

Clinical details, miRNA mature strand expression RNAseq (HiSeq) and gene expression RNAseq (HiSeqV2 from the Cancer Genome Atlas (TCGA)) were from two different cohort studies. Colon adenocarcinoma (COAD) and rectal adenocarcinoma (READ) were extracted to analyze miR-330-3p, miR-330-5p, and BACH1 expression between tumors and adjusted normal and survival analysis^24^; then, samples were combined into the COADREAD cohort. Both BACH1 mRNA and miR-330-3p and miR330-5p expression levels were processed as depicted in our past reports.^25,26^ They were shown as log2 (RPM+1) and expression levels were compared with each other through using Mann-Whitney U tests. Overall and recurrence-free survival curves were generated for high and low expressions as described previously,^27^ utilizing the median level of expression as the cut-off and log-rank tests for statistical analyses.

### Cell lines and construct transfection 

Human CRC cells (HCT116 and SW480) were cultured in RPMI 1640, containing 10% FBS (GIBCO, Carlsbad, CA, USA) at 37°C with 5% CO_2_ condition. The cells were obtained from Pasture institute of Iran (Tehran, Iran) and all experiments were done in cell logarithmic phase. 2×10^5^ HT116 and SW480 cells were transfected by PCMV-miR-330 and empty PCMV construct (as a negative control) at 50%-60% confluence. In order to transfect the constructs into the cells, jetPRIME transfection reagent (Polyplus, France) was used to transfer of PCMV vector (Origene, USA) to the cells according to the company’s instructions. The PCMV construct contains Geneticin resistant sequences for selecting the plasmid recipient cells. The selection of vector positive cells was done in two weeks by using geneticin antibiotic (50 mg/mL).

### Gene expression analysis

In order to evaluate the BACH1 mRNA and miR-330 level, total RNA was purified from CRC cells using RiboEX reagent (GeneAll, GeneAll biotechnology, Seoul, Korea). In order to cDNA synthesis for BACH1, BioFact cDNA synthesis kit was used for the synthesis of 1 µg of total RNA (Daejeon, Korea) via Boi-Rad thermal cycler instrument (Hercules, CA) according to manufacturer’s instruction. The reactions for qRT-PCR assays contained 5 µL of 2X SYBR green master mix (Biofact, Daejeon, Korea), 0.25 µL of specific primer (Table 1), 0.5 µL of cDNA, and 4.25 µL of distilled water. The mixture amplified using LightCycler 96 instrument (Roche Diagnostics, Mannheim, Germany).

**Table 1:** Specific Primers Sequences

**Name**		**Sequences**
Beta-Actin	F	5’- TCCCTGGAGAAGAGCTACG -3’
	R	5’- GTAGTTTCGTGGATGCCACA-3’
BACH1	F	5’-TGCGATGTCACCATCTTTGT-3’
	R	5’-CCTGGCCTACGATTCTTGAG-3’
U6 snRNA	F	5’- CTTCGGCAGCACATATACTAAAATTGG -3’
	R	5’- TCATCCTTGCGCAGGGG -3’
miR-330	F	5’- TCTCTGGGCCTGTGTC -3’
	R	5’- CCAGTTTTTTTTTTTTTTTGCCTAAG -3’

The miScript II RT Kit (Qiagen) was used to synthesize cDNA for the miRNA evaluation. The qRT-PCR for miR-330 were performed by Exiqon SYBR green master mix and specific primers (Table 1) for studying the microRNA expression. Beta-actin and U6 were used as internal control for BACH1 and miR-330, respectively.

### Colorectal cancer proliferation assay

To identify the regulatory role of miR-330 on the CRC cells proliferation, MTT assay (Sigma-Aldrich, St. Louis, MO) was performed. Briefly, 10×10^3^ of the stable cells were seeded in 96-well culture plate. 150 µL of RPMI 1640 contained 10% FBS and 2 mg/mL of MTT were added to the cells 24 hours later.

Then, the cells were incubated in a cell culture condition at dark condition for 4 h. After that, 100 µL DMSO and Sorensen buffer (4:1 V: V) were added to each well to dissolve the formazan crystals (purple) and shook for 2 minutes at 1000 rpm. Finally, the wells quantified at 570 nm using an ELISA microplate reader (Sunrise; Tecan Co., Austria).

### Western blot

#### Protein extraction

Total cellular protein was isolated from the CRC cells using Santa Cruz protein lysis buffer (RIPA, Santa Cruz Biotechnology, CA). Protein lysis buffer, PSMF, a phosphatase inhibitor, and protease inhibitor were added to the cell pellet. After vortexing and incubation on the ice, total proteins were isolated by centrifugation.

#### SDS-PAGE and blotting

Fifty micrograms of total protein were separated by 10% SDS-PAGE. The obtained proteins were transferred into the polyvinylidene difluoride membrane via the semi-dry blotting method. After that, the membrane was blocked via blocking buffer (phosphate buffer contained 0.5% Tween 20) on a shaker at 25°C for 2 hours. Then, they were incubated with primary mouse monoclonal antibodies against BACH1, CXCR4, MMP9, and VEGFR (1:1000, Santa Cruz Biotechnology, Ca) overnight at 4°C. Beta-actin was used as a reference protein. Then, the membranes were incubated with HRP conjugated secondary antibody (rabbit anti-mouse) (Bio‐Rad, Hercules, and CA) (1:5000, diluted in PBS). Finally, the obtained protein bands were developed under the electrochemiluminescence system (Roche, Germany). Subsequently, the bonds imaged by western blot recording instrument (Sabz.co, Iran). The bonds quantified using ImageJ software (http://imagej.nih.gov/ij).

### Statistical analysis

The results of different experiments were expressed as the means ± standard deviation (SD). The results were analyzed by GraphPad Prism (San Diego, CA) software. The p-value of parametric results was calculated by one-way and two-way ANOVA. Nonparametric results, including clinical assays that were extracted from TCGA database were analyzed by Mann-Whitney U test. A *P* value <0.05 was considered as statistically significant.

## Results and Discussion

### The miR-330 downregulation and BACH1 up-regulation are associated with poor CRC patient survival

**Figure 1:**
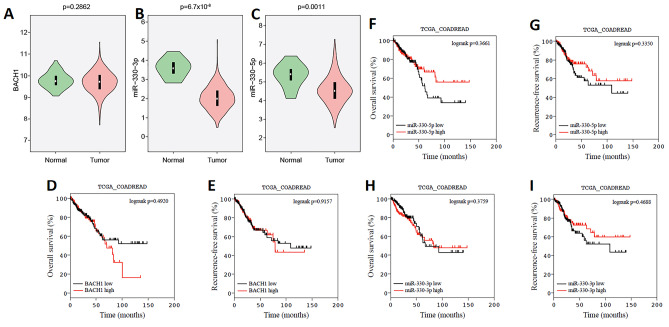
**BACH1 overexpression, and miR-330 down-regulation in human CRC and overall survival (OS) and recurrence-free survival (RFS) of CRCs with high and low BACH1, miR-330-3p and miR-330-5p expressions in tissues. ** The expression of BACH1 (A), miR-330-3p (B) and miR-330-5p (C) were analyzed with the data of CRC and their adjusted tissues, which were extracted from the TCGA database. Kaplan–Meier curves were used for RFS and OS analyses between the high and low levels of BACH1 (D and E), miR-330-5p (F and G), and miR-330-3p (H and I), in CRC. The log-rank test is used.

The statistical analysis showed the overexpression of BACH1 in tumors compared to the adjacent normal tissues (Figure 1A). In addition, the results of miR-330-5p and miR-330-3p expressions showed their down-regulation in CRC tumor tissues compared to the colorectal normal tissues (p=0.0011 and p=6.7×10-^4^, respectively) (Figure 1B, C). More than 300 patients with CRC were included in overall survival (OS) and recurrence-free survival (RFS) analyses. The patients’ clinical data and BACH1, miR-330-3p and miR-330-5p expressions were extracted from the TCGA database. The results of the survival analysis showed that there is not a significant difference in OS and RFS for the patients whose tumors express low and high levels of BACH1 (Figure 1D, E). In addition, we did not detect any significant differences in OS or RFS in patients with low versus high miR-330-3p and/or miR-330-5p expressions (*P* > 0.05) (Figure 1F-I).

Studies confirmed miRNA as a major regulator in cellular processes especially in cancer development. In 2004, Calin and colleagues showed that half of the microRNAs genes (52.5%) are situated at cancer-related genomic regions.^28^ The miR-330 was firstly discovered by Weber in 2005.^29^ Gaur et al in 2007 reported that miR-330 down-regulated in tumors.^30^ Li et al showed that miR-330 regulated the Cdc42 negatively. They further discovered that miR-330 could induce apoptosis, G1 cell cycle arrest, and anti-proliferation in CRC cells.^31^ Altogether, these studies confirmed the miR-330 downregulation in most types of cancer such as CRC and could act as TS-miRNA.

The results of TCGA clinical analysis revealed that miR-330 was reduced in CRC tissues compared to normal tissues.

A study on colon cancer implied that BACH1 suppression by siRNA could notably prevent colon cancer cell (HT-29) migration. They also demonstrated higher expressions of BACH1, CXCR4, and MMP1 in the colorectal cell line (HT-29) and CRC tissues compared with the normal controls. They suggested high expression levels of BACH1 may be correlated with the distant metastasis of CRCs.^32^ The results of survival assays from the TCGA database illustrated that BACH1 overexpression could involve in overall survival and poor recurrence-free patient survival.

The present study showed that there is a negative correlation between BACH1 and miR-330 in the CRC.

### The miR-330 could target BACH1 expression

**Figure 2:**
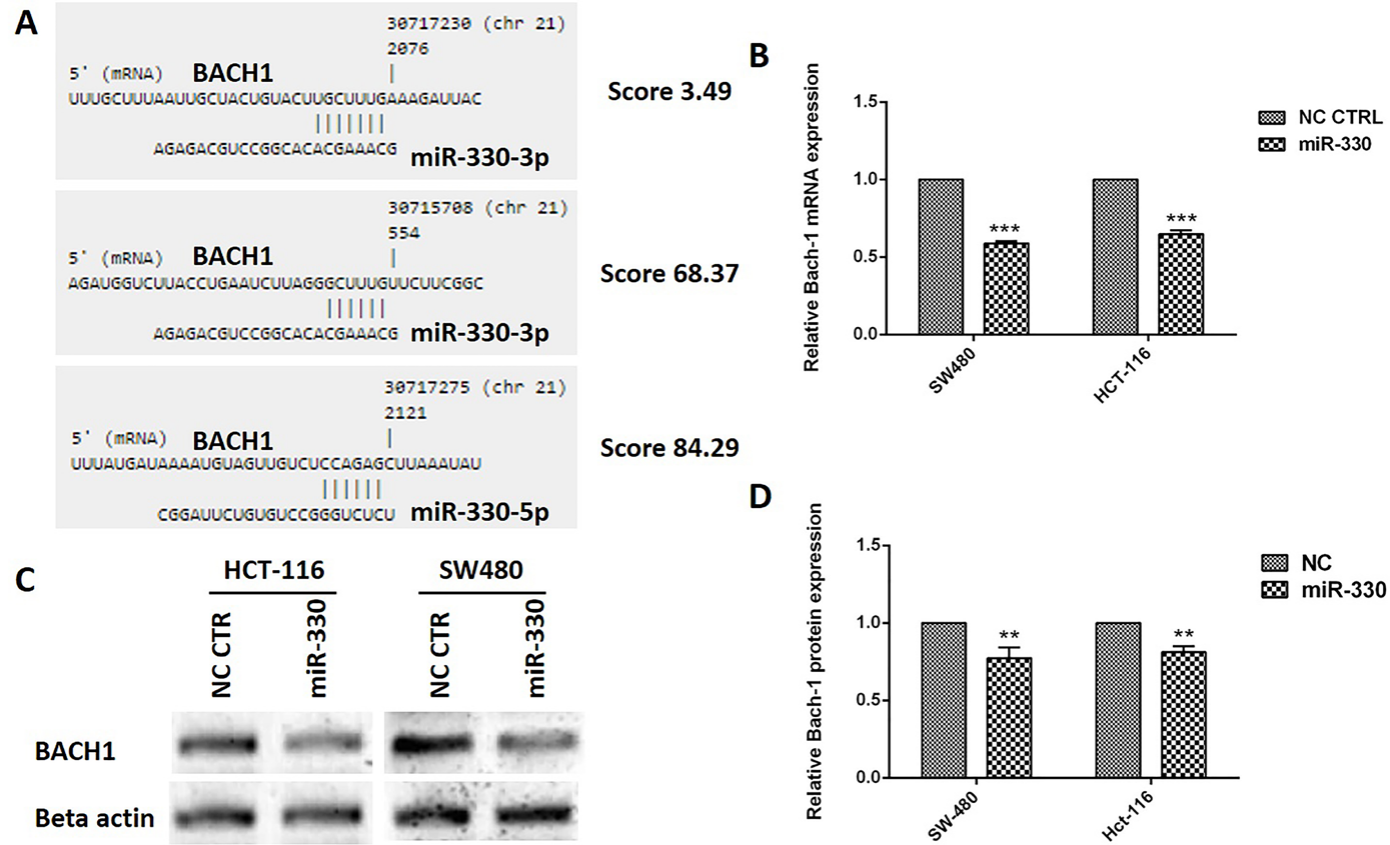
**The miR-330 could target BACH1 mRNA in CRC cells.** The bioinformatics analyses showed that miR-330-3p targets BACH1 mRNA from two regions and miR330-5p targets this mRNA from one side (A). The qRT-PCR analysis showed that miR-330 induction could reduce the BACH1 mRNA expression in both colorectal cell lines (B). The BACH1 protein level was reduced in the miR-330 replaced cells (C-D). The data represent as mean±SD (n=3); ** *P*  < 0.01 and *** *P*  < 0.001 versus negative control (NC) cells.

Bioinformatics analysis according to the protocol of Vejnar et al^33^ revealed that miR-330-3p could directly bind to 3’UTR of BACH1 mRNA from two sides with the prediction score of 3.49 and 68.37. In addition, the software predicted miR-330-5p could target the 3’UTR of BACH1 mRNA with 84.29 score (Figure 2A). The gene expression analysis showed that BACH1 was reduced to 0.59 ± 0.01 and 0.65 ± 0.02 in the miR-330 induced SW480 and HCT116 cells, respectively (Figure 2B). Besides, the western blot analysis for BACH1 protein showed that miR-330 could decrease the protein expression to 0.77 ± 0.07 and 0.81 ± 0.04 in SW480 and HCT116 cells, respectively (Figure 2C, D).

The hypothesis is that a decrease in miR-330 levels can result in the dysregulation of BACH1. In addition, the bioinformatics analyses further suggested that both miR-330-3p and miR-330-5p could target BACH1 mRNA. Similarly, it was shown that both BACH1 mRNA and protein levels were reduced after miR-330 induction into the CRC cell lines.

### The miR-330 decreased the CRC cells proliferation

**Figure 3:**
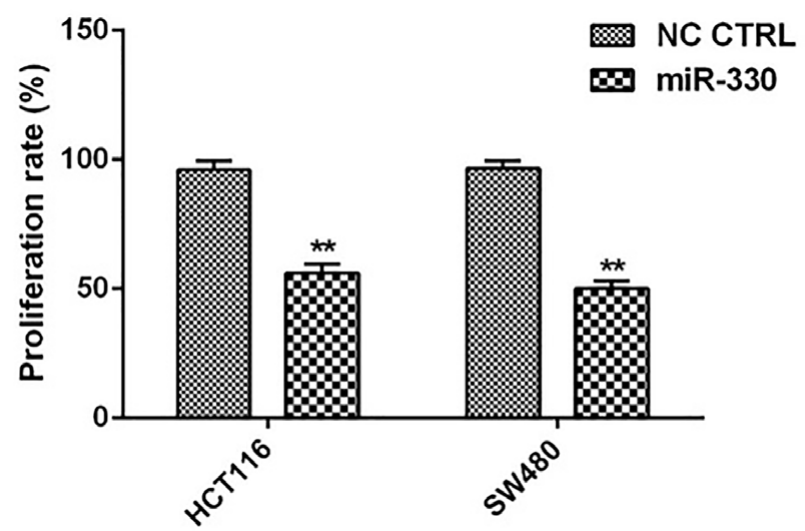
**Modulation of CRC cell proliferation by miR-330.** The percentage of the proliferated cells was shown after 48 h. The data represent as mean±SD (n=3); **P <0.01, versus negative control (NC) cells.

After replacement of miR-330 into HCT 116 and SW480 cells, proliferation assay performed by MTT assay showed that the replacement of miR-330 in CRC cells inhibited 56 ± 3.6% and 50 ± 3% of the proliferation rates in HCT116 and SW480 cells, respectively (Figure 3).

Mao et al, in line with our study, identified miR-330 as anti-proliferative miRNA in prostate cancer. They found the inverse association between the miRNA and specificity protein 1 (Sp1), the proliferation-associated gene. In another study, Su et al suggested that upregulation of miR-330-5p suppressed the proliferation of malignant melanoma cells in vitro via regulation of PDIA3-dependent pathways and DIA3 expression involved in maintaining the hair cycle.^34^ Our previous study also showed that miR-330 could inhibit CRC migration.^35^ According to those results, miR-330 inhibited migration and invasion by targeting Sp1 mRNA and following reduced the expression of MMP2 and MMP9 in prostate cancer cells.^36^ In another research, Tréhoux et al demonstrated that miR-330 along with miR-29a could suppress the pancreatic cancer cells development and their invasion by targeting Mucin 1 (MUC1). Similarly changes in the glycosylation of MUC1 has been linked with carcinogenesis. They also demonstrated dual miR-330 and miR-29a induction sensitization to gemcitabine *in vitro*. They also confirmed anti-tumoral activity of these two miRNAs in xenografted pancreatic tumors.^37^

### The miR-330 modulated MMP9, CXCR4, and VEGFR proteins expression in CRC cells

**Figure 4:**
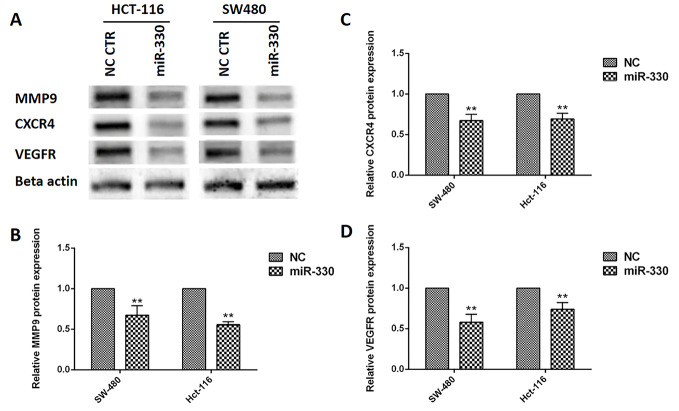
**MMP9, CXCR4, and VEGFR proteins expression after miR-330 induction in CRC cell lines.** MMP9 (A and B), CXCR4 (A and C), and VEGFR (A and D), proteins expressions were evaluated by western blot. Data are presented as means ± SD. (n=3); ** *P* <0.01 versus negative control (NC).

To understand the association between the miR-330 and BACH1 suppression on oncogenic and EMT-related proteins, western blot analyses performed on MMP9, CXCR4, and VEGFR proteins (Figure 4A). The relative proteins expressions were 0.67 ± 0.11 and 0.55 ± 0.04 for MMP9 (Figure 4B), 0.67 ± 0.07 and 0.69 ± 0.07 for CXCR4 (Figure 4C), and 0.58 ± 0.01 and 0.74 ± 0.08 for VEGFR (Figure 4D), respectively.

**Figure 5:**
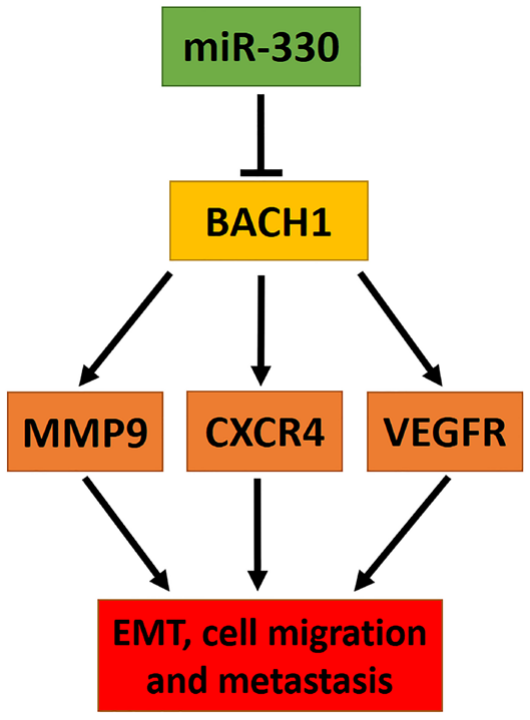
**Model of miR-330 regulation by modulating BACH1 and the proteins involved in its downstream.** miR-330 by targeting BACH1 in posttranscriptional level reducing EMT related proteins, including MMP9, CXCR4, and VEGFR levels cooperated to the reduced cell migration and metastasis.

The findings of this study suggested that miR-330 could regulate the major proteins (VEGFR, MMP9, CXCR4) involved in cancer cell epithelial-mesenchymal transition (EMT), migration, and metastasis by modulation of BACH1 (Figure 5).

The studies also described that multiple biomolecules such as chemokines, cytokines, growth factors, and MMPs have a major role in metastatic cancer.^38,39^

The evidence suggested chemokines and their receptors are involved in homing and proliferation of metastatic tumor cells at new *homing* sites. It was shown that CXCR4 upregulated in human metastatic tumors, for instance, oral squamous cell carcinoma, CRC, prostate cancer, and breast cancer.^40-42^ Muller et al demonstrated that CXCR4 has an important signaling factor in the mobility of metastatic breast cancer via increasing chemotactic responses.^43^

Vascular endothelial growth factor (VEGF) has been well identified as a critical regulatory factor in tumor growth and stimulates the angiogenesis via inducing migration and proliferation of endothelial cells. High-level expressions of VEGF has been revealed in numerous types of human malignancies.^44^ The cascade of signal activated when VEGF bind to the VEGFR. The binding of ligand with its receptor causes activation of signaling pathways including phospholipase Cγ1 (PLC γ1), a mitogen-activated protein kinase pathway via Ras/Raf1 activation, and phosphoinositide 3-kinases (PI3K)/protein kinase B (Akt) pathway.^45^ In addition, Kryczek et al reported that stimulation of CXCR4 accompanied by increasing in VEGF secretion lead to enhanced angiogenesis of ovarian cancers and metastasis.^46^

MMPs belong to proteinase family that is capable of degrading the extracellular matrix proteins and subsequently affect tumor behavior. MMP-9 and MMP-2 are one of the key molecules for tumor invasion and metastasis.^47^ Elevated MMP9 plasma activity and protein concentration were demonstrated in prostate cancer patients^48^ and correlated with prostate cancer bone metastasis.^49^

## Conclusion

In conclusion, we observed that miR-330 is down-regulated in CRC. Replacement of miR-330 decreased cell proliferation and migration via down-regulation of the proteins involved in cell proliferation and migration in CRC cells. We also witnessed that this might occur via targeting of BACH1. It is believed that the restoration of miR-330 may bring a promising approach for the treatment of CRC patients.

## Ethical Issues

Not applicable.

## Conflict of Interest

All Authors declare no conflict of interest.

## Acknowledgements

This work was financially supported by the Iran national science foundation [grant number 93041394].
